# Prediction of COVID-19 Pandemic in Bangladesh: Dual Application of Susceptible-Infective-Recovered (SIR) and Machine Learning Approach

**DOI:** 10.1155/2022/8570089

**Published:** 2022-04-26

**Authors:** Iqramul Haq, Md. Ismail Hossain, Ahmed Abdus Saleh Saleheen, Md. Iqbal Hossain Nayan, Mafruha Sultana Mila

**Affiliations:** ^1^Department of Agricultural Statistics, Sher-e-Bangla Agricultural University, Dhaka 1207, Bangladesh; ^2^Department of Statistics, Jagannath University, Dhaka 1100, Bangladesh; ^3^Quality Services and Compliance, Square Pharmaceutical Limited, Dhaka, Bangladesh

## Abstract

The outbreak of COVID-19 is a global problem today, and, to reduce infectious cases and increase recovered cases, it is relevant to estimate the future movement and pattern of the disease. To identify the hotspot for COVID-19 in Bangladesh, we performed a cluster analysis based on the hierarchical k-means approach. A well-known epidemiological model named “susceptible-infectious-recovered (SIR)” and an additive regression model named “Facebook PROPHET Procedure” were used to predict the future direction of COVID-19 using data from IEDCR. Here we compare the results of the optimized SIR model and a well-known machine learning algorithm (PROPHET algorithm) for the forecasting trend of the COVID-19 pandemic. The result of the cluster analysis demonstrates that Dhaka city is now a hotspot for the COVID-19 pandemic. The basic reproduction ratio value was 2.1, which indicates that the infection rate would be greater than the recovery rate. In terms of the SIR model, the result showed that the virus might be slightly under control only after August 2022. Furthermore, the PROPHET algorithm observed an altered result from SIR, implying that all confirmed, death, and recovered cases in Bangladesh are increasing on a daily basis. As a result, it appears that the PROPHET algorithm is appropriate for pandemic data with a growing trend. Based on the findings, the study recommended that the pandemic is not under control and ensured that if Bangladesh continues the current pattern of infectious rate, the spread of the pandemic in Bangladesh next year will increase.

## 1. Introduction

Novel coronavirus 2019 (nCoV-2019) is the world's third zoonotic human virus of the century, which is comparable to previous infections such as severe acute respiratory syndrome coronavirus (SARS-CoV) and Middle East respiratory syndrome coronavirus (MERS-CoV), which spread to 37 and 27 countries in 2002 and 2012, respectively [1–4]. After its emergence in Wuhan City, Hubei Province of China, on December 31, 2019, it has spread to more than 200 countries worldwide, according to the World Health Organization (WHO), and the world has had 202,370,504 confirmed cases up to August 6, 2021, where the fatality rate was 2.1% [5]. COVID-19 was declared a pandemic by the World Health Organization (WHO) on March 11, 2020, due to its enormous spread [6, 7]. The first case of COVID-19 was detected on March 8, 2020, in Dhaka by two men who were Italian returnees, and the woman was a family member of one of these two men. This was followed by the diagnosis of an average of 4–10 cases in the following days, which began to increase rapidly from April 2020 and now exceeded 13,35260 confirmed cases by August 6, 2021 [6, 8] (Institute of Epidemiology [9]).

COVID-19 is a pandemic and it will not be controlled until the vaccination rate increases. Globally, up to August 6, 2021, only 15.8% of the total population has been vaccinated, while only 1.2% of people in low-income countries have received at least one dose of the vaccine, and this rate is gradually increasing [10]. Most people were careless about the pandemic in a densely populated country like Bangladesh. The future direction of the COVID-19 trend has a significant impact not only on people's health issues but also on economic stability. The daily numbers of confirmed cases, active cases, death cases, and recovered cases rapidly fluctuate in Bangladesh.

To prevent and manage the spread of COVID-19, various models have been used to predict how the virus spreads across populations, such as SI (susceptible-infected), SIR (susceptible-infected-recovered), and SIS (susceptible-infected-susceptible) [11]. In the past, various researchers tried to use various epidemiological models to predict epidemiological conditions. Dantas, Tosin, and Cunha Jr. [12] attempted to use the SEIR-SEI model to describe the Zika virus outbreak in Brazil. Another researcher was studying the effectiveness of modeling methods during the pandemic and using information from COVID-19 to develop an SIR model [13]. Yang *et al.* [14] and Lai *et al.* [15] tried to predict the trend of the COVID-19 pandemic using the SEIR model, which is a commonly used mathematical epidemic model that describes the distribution of an epidemic. Wang et al. [16] also used the SEIR model (revised SIR model) to predict the situation of the COVID-19 pandemic, which then had a positive impact on the prediction of the proposed government policy. These models clearly provide a more detailed description of the epidemic spread but their identification is also significantly harder. One of the reasons is that they are usually characterized by a large number of parameters and variables, while the data is usually noisy and in limited quantities, a situation that makes these models prone to overfitting. Then, considering the characteristics of the data of this study, our research presents the simple epidemic model, that is, SIR (susceptible-infective-recovered) method, based on the predictive model of epidemic phenomena, which is one of the most widely accepted statistical models for forecasting inherent contagion scenarios and is widely used to assess infection data during the several stages of an epidemic outbreak [17, 18]. It is based on a series of dynamic ordinary differential equations that take into account the number of people affected by the infection, as well as the trend over time of people recovering after infection [11].

In the current pandemic, to model and predict new COVID-19 infections, deaths, and recoveries, several researchers have used different techniques, including statistical, mathematical, machine learning, and deep learning algorithms [19, 20]. A study conducted by Papastefanopoulos et al. [21] compared various statistical and machine learning time series models, such as ARIMA, PROPHET, Holt-Winters additive model, TBAT, Deep-AR, and N-Beats, to forecast COVID-19 cases. In this study, we used the Facebook PROPHET Forecasting Procedure for COVID-19 forecasting. Although PROPHET is considered a new approach, it is renowned for its easy-to-use yet powerful model [22]. There are several studies that use the Facebook PROPHET Forecasting Procedure [23, 24]. Based on confirmed cases, cluster analysis techniques were used to determine if there are homogeneous groups among districts.

All compartmental models, such as SIR, SIRD, and SEIR, are usually defined by constant parameters. Time-invariant parametric models may be unable to capture the consequences of such changes, resulting in poor prediction results [25]. Furthermore, the data utilized to construct the SIRD model varies by location, resulting in unreliable predictions [26]. SEIR is a complex model which is less accurate in making prediction when a larger number of parameters are included in the model. When compared to the SEIR model, SIR model has shown greater predictive ability in terms of number of instances predicting [27]. The SIR model is thought to be more capable of long-term forecasting than the regression model. Researchers used SIR model and machine learning that provide smart healthcare for prediction as well as prevention of COVID-19 [11], and simple epidemic models may provide more concise and accurate description of the spread of COVID-19 than complex models [28]. So, based on the literature, the main objective of this study is to identify the future direction of the COVID-19 trend in Bangladesh based on an epidemiological model called the SIR and Facebook's PROPHET Procedure using data from the Institute of Epidemiology Disease Control and Research (IEDCR) from 1st April, 2020, to 6th August, 2021. However, a few studies have used the comparison between machine learning algorithms and epidemic models to identify the best predictive models for the future direction of the COVID-19 scenario worldwide. To the best of our knowledge, the originality of the study is that it is almost new in the field of the future direction of the COVID-19 pandemic in the Bangladeshi context, for the first time using two different methods (epidemic model and machine learning method) to compare the future direction of the COVID-19 pandemic in Bangladesh, which will assist future data scientists. In addition, the study also aims to determine the best model to forecast the trend of the COVID-19 pandemic, which may help decision-makers understand the future direction of COVID-19 in Bangladesh. This study also used cluster analysis to determine the homogeneity between districts based on the confirmed cases of COVID-19 in Bangladesh, which may reveal where medical improvements are still needed to control the epidemic situation.

## 2. Materials and Methods

### 2.1. Data Source

The analysis was performed on the information gathered through an open-access data set and the IEDCR site of IEDCR in Bangladesh [9]. They discharge official information as they gather information from the distinctive authority clinical parts of COVID-19 patients' treatment. After collecting IEDCR data (confirmed, death, and recovery cases), we compared it with other access points (such as Worldometer, Directorate General of Health Services (DGHS) dashboard) to obtain a more consistent quality of the COVID-19 data. The first case of COVID-19 was detected in Bangladesh on March 8, but the pandemic cases grew rapidly in April, so we consider the IEDCR data from 1 April 2020 to 6 August 2021 [9]. Since our participants were patients, those who were engaged with COVID-19 confirmed cases, death cases, and recovered cases from 1 April 2020 to 6 August 2021 in Bangladesh, there were no other exclusion criteria. During the study period, only 2.7 percent of the population in Bangladesh received the second dose of the COVID-19 vaccine [29]. As of August 6, 2021, the number of confirmed cases for males represents 65% and that of females represents 35%. Furthermore, males account for 67% of COVID-19 deaths, while females account for only 33%.

### 2.2. Analytical Techniques

In this study, we attempt to utilize a notable epidemiological model, SIR, to describe the spread of the disease and compute the number of infected and dead individuals with three subclasses of the population to predict the COVID-19 direction in Bangladesh. Some recent studies have tried to predict the risk of coronavirus by applying the SIR model in Bangladesh and elsewhere [30–32]. Here, the total population is denoted by *N*, which is further subdivided into three subclasses to simulate the epidemic of COVID-19, which are the susceptible (*S*), the infected (*I*), and the recovered (*R*). People who develop the disease in a susceptible condition still belong to subclass *S*. Those who are infected as well as those who have the capability of transmitting the disease are in subclass *I*. Finally, the individuals who are immune are included in subclass *R* [32, 33].

In SIR modeling process, the study independent variable is time which is measured as days. There are two types of dependent variables: (a) number of people in each of the groups (*S*, *I*, and *R*) and (b) fraction of the total population in each of the three categories. The model is then governed by the following set of nonlinear differential equations:

(1) $$\begin{array}{c} \frac{dS}{dt}=-\frac{\beta IS}{N}, \end{array}$$

(2) $$\begin{array}{c} \frac{\text{d}I}{\text{d}t}=\frac{\beta IS}{N}-\gamma I, \end{array}$$

(3) $$\begin{array}{c} \frac{\text{d}R}{\text{d}t}=\gamma I. \end{array}$$

Here, equation (1) is for susceptible compartments, equation (2) is for infectious compartments, and equation (3) is for recovered compartments. These differential equations are dependent on time (days in this case) and solved for any inputs of *N*, *β*, and *γ* and initial values of *S*, *I*, and *R*. Here the nature of these differential equations is ordinary because of all the differential equations involving ordinary derivatives, that is, derivative with respect to single independent variable. To solve these nonlinear ordinary differential equations for an epidemic, start with *I*, which must be greater than zero, and then increase exponentially. The fundamental reproductive ratio (*R*_0_) that can be calculated using the estimate is another main component, and the formula is *R*_0_=(*β*/*γ*). Here, *β* is the infectious rate and *γ* is the recovery rate. The infection rate is the number of people infected at a given time, and the recovery rate is the number of people who go from being infected to having recovered at a given time. If infectious rate  <  recovery  rate, then we conclude that the pandemic will die out. If infectious rate > recovery rate, then we conclude that the pandemic will spread over time. For more information about the model, we suggest that the reader read these papers [13, 33, 34].

“PROPHET” is a time-series data forecasting method created by the Facebook Core Data Science team. According to Facebook, PROPHET is most suitable for time series with strong seasonal influence and multiple seasons with historical data and resistance to outliers and trend changes. In this case, our data has no seasonality, but it works well, and this is what PROPHET is good for. To perform analysis, we must first prepare our data set and create a data frame with two columns named “ds,” that is, date frame, and “y,” which is the case we want to predict [35]. Cluster analysis was performed using “hierarchical *k*-means” using the *R* package named “cluster.” Cluster analysis is widely used in social science and commercial market research, as well as epidemiology and public health research [36–38]. This unsupervised learning is usually used to discover hidden structures in the data where the correct group is not known in advance. It helps to find groups or natural patterns in the data so that items in one group are more similar than items in another group.

In light of the accessible information and the SIR suspicions, an *R* Project for statistical computing version 4.0.0 with package “deSolve” was used to make the SIR model. For the graphical area, “ggspatial” and “ggplot2” were used for better illustration. The shapefile for geographical mapping was downloaded from “https://data.humdata.org/dataset/administrative-boundaries-of-bangladesh-as-of-2015,” which is publicly available.

## 3. Results and Discussion

### 3.1. Geographical Illustration

**Figure 1:**
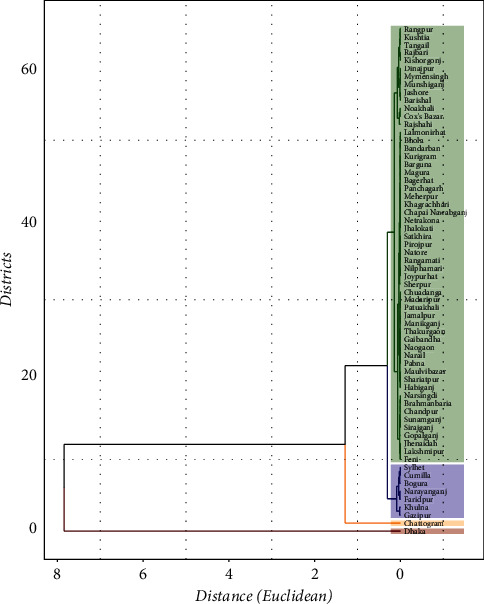
Cluster of districts based on COVID-19 cases.

Bangladesh has now faced the second wave of COVID-19. The second wave of the COVID-19 epidemic is currently in a better phase in Bangladesh [39]. The recent update of COVID-19 cases in Bangladesh observed that some districts are more at risk than others. The hierarchical *k*-means clustering is shown in the dendrogram in Figure 1, where each district corresponds to one object. All of the sixty-four affected (number of confirmed cases) districts in Bangladesh were used to construct clusters of homogeneous districts. Four clusters of sizes 1, 1, 7, and 55 were formed with boxes around clusters of districts having a similar number of active positive cases of COVID-19, as shown in the diagram (Figure 1). Here, cluster 1 has only Dhaka district, cluster 2 has only Chattogram district, and cluster 3 has Bogura, Sylhet, Gazipur, Narayangonj, Cumilla, Faridpur, and Khulna districts, and the rest of the districts are in cluster 4. We displayed Figure 1 horizontally, where the *y*-axis indicates the dissimilarity/distance between two objects (i.e., districts) and the *x*-axis indicates the districts of Bangladesh. The greater the distance between objects is, the less similar the objects are.

**Figure 2:**
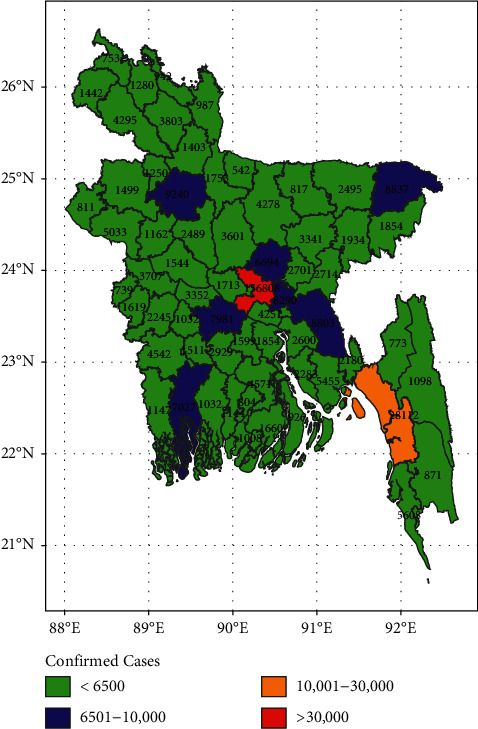
The COVID-19 hotspot map based on the clustering of districts regarding COVID-19 cases in Bangladesh.

Figure 2 shows that Dhaka was marked as the first cluster, which indicates that Dhaka is now a hotspot for COVID-19 in Bangladesh. The red spots indicate that the highest number of confirmed cases of COVID-19 was in Dhaka City. Dhaka is the capital and has the largest population density. Visitors from other countries arrive here first. More people were oblivious to the dangerous situation and continued to work in factories or industries until late March. This is one of the reasons for the extremely high number of positive cases in Dhaka city [40].

Chattogram was considered as the second cluster, which indicated that it was also a high-risk zone for the COVID-19 pandemic. In a separate study from Bangladesh, researchers observed that six districts, including Dhaka, were recognized as significant hotspots for the COVID-19 pandemic, while Chattogram was an expanded contaminated area, indicating the virus' slow spread to distant districts [41].

However, Bogura, Sylhet, Gazipur, Narayangonj, Cumilla, Faridpur, and Khulna districts were listed as the third cluster in this analysis, and it was reported that these districts were also a medium-risk zones for the COVID-19 pandemic. Other districts (green) were considered the fourth group in this analysis, and it was implied that these districts were a low-risk zone for COVID-19 for Bangladesh (Figure 2).

### 3.2. Prediction with the SIR Model

We used the mathematical and compartmental model to predict the COVID-19 situation in Bangladesh. The average infectious life time was 22 days (1/*γ*=22 days) [42, 43], and we obtained *β*= (0.095445/day) and *γ*=0.04545. Here, the basic reproductive ratio *R*_0_=2.10 > 1, which implies that the infection rate is greater than the recovery rate, and the average number of people infected by one other person is greater than 2 in this analysis. That is, *R*_0_ measures the spread of an infectious disease [44]. Masuda et al. [42] found that the basic reproduction number *R*_0_ in Bangladesh was between 1.5 and 12. It is significantly better than other pandemic viruses like SARS/MARS [45]. The result of the basic reproduction ratio is compatible with western Europe and the United States [44, 46].

**Figure 3:**
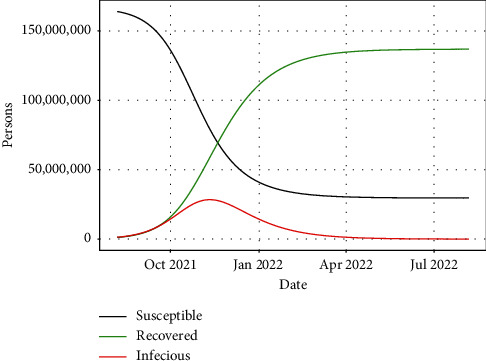
Prediction of the COVID-19 outbreak in Bangladesh based on the SIR model (red color = infectious/confirmed cases, black color = susceptible, and green color = recovered cases).

From Figure 3, we observe that the pandemic would decline in Bangladesh by August 2022. This is support for the earlier study conducted in Bangladesh and implies that if the situation in Bangladesh remains the same, by December 2022, the pandemic would be over [40]. In terms of recovered cases, the number of recovered people has increased since October 2021 (Figure 3). A previous study in Bangladesh by applying the SEQIRP model (modified SIR model) observed that, after 400 days (April 2021), the actual recovery rate was increasing, with a value of roughly 0.56 [47].

### 3.3. Forecasting Confirmed, Death, Recovered, and Active Cases in Bangladesh

**Figure 4:**
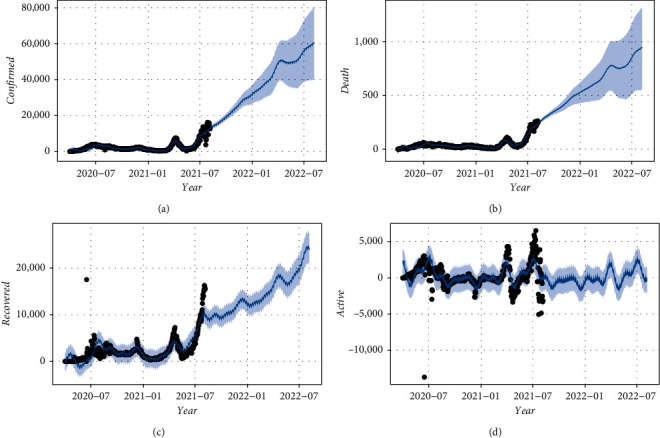
Time-series forecast plot using Facebook's PROPHET Procedure for the COVID-19 outbreak in Bangladesh. Confirmed cases (a), death cases (b), recovered cases (c), and active cases (d).

Figure 4 indicates the trend of cases from 1st April, 2020, to 6th August, 2021 (in black dots) and thus predicts the trend for the next 12 months, August 2021 and August 2022 (in blue dots), using the time-series forecasting approach.

Figure 4(a) shows the curve of the confirmed cases from April 1, 2020, to August 6, 2021, and shows the time-series prediction curve for the next 12 months (August 2021 and August 2022) in Bangladesh. Here, the *x*-axis shows months and the *y*-axis shows confirmed cases in Bangladesh. According to the recent report of IEDCR, the first delta variant cases of COVID-19 infection among Bangladeshi citizens were identified on May 8, 2021 [9]. If the current scenario of COVID-19 in Bangladesh remained the same, the confirmed cases in Bangladesh would have increased exponentially in the coming years of August 2021 and August 2022, and we also remark that the cases have been increasing since July 2021 (Figure 4(a)). The finding of this study is consistent with an earlier study conducted in three countries, namely, Australia, Italy, and the UK, and indicates that the growth rate of confirmed cases will increase in the next year [48]. Another similar study conducted by Mahanty and team members observed that India, USA, and Brazil's growth rate of confirmed cases is increasing day by day, and the rate tends to be exponential [49]. In contrast, Hossain et al. [50] showed that confirmed cases in Bangladesh gradually decreased for the next 40 days.

Figure 4(b) shows the curve of the death cases from April 1, 2020, to August 6, 2021, and shows the time-series prediction of the death curve for the next 12 months (August 2021 and August 2022) in Bangladesh. According to Figure 4(b), we found that death cases in Bangladesh will also increase exponentially in the coming years of August 2021 and August 2022, and we also notice that these death cases have been increasing since July 2021 in Bangladesh. This result is not consistent with the earlier studies conducted in Bangladesh [50] and Saudi Arabia [51], which revealed that death cases were constant for the next 40 days in Bangladesh and forecast no death cases for the coming days in Saudi Arabia.

Figure 4(c) shows the curve of the recovered cases from 1 April 2020 to 6 August 2021 (black dots), and we undertake a time-series forecasting approach and predict the number of recovered cases between August 2021 and August 2022 (blue line) in Bangladesh. According to Figure 4(c), we found that recovered cases in Bangladesh also increased dramatically from August 2021 to August 2022, which indicates that these cases also increased in July 2021 in Bangladesh. During the study period between March and October 2020, the number of recovered cases increased every day in Saudi Arabia [51].

Figure 4(d) shows that, at the end of August 2021, there was an increase in the number of active cases in Bangladesh; PROPHET plotted the observed values of our time-series (the black dots) and the forecasted values (the blue line) from August 2021 until August 2022. In terms of active cases of COVID-19 patients in Bangladesh, we can see that the curve is in either an upward or downward direction. As you can see, the curve is going down compared to the confirmed curve and the death curve, which is a very beautiful sign for Bangladesh.

## 4. Limitations

Because prediction is a numbers game, it is not always accurate. Although SIR model-based prediction is widely accepted, it cannot provide an accurate estimate. However, if unexpected changes occurred in COVID-19 transmission patterns, they would have an impact on these predictions. This study inherits all the limitations of the SIR model. The prediction model can be used to understand how an infectious disease spreads. Even if predictions are inaccurate, they have been producing essential knowledge about epidemic situations and how to stop the spread of the virus. Data quality is also an important issue in this case [52]. We need to develop a proper model and assumptions based on real data, enact rigorous lockdown procedures, and quarantine all COVID-19 carriers. The predictions were made without considering the socioeconomic conditions and environmental conditions that potentially might affect the prediction curve in this analysis. Various studies, for example, show that there was a positive association between air pollution and spread of the virus and implies that higher mortality was found to be linked to poor air quality, namely, high PM2.5, carbon monoxide, and nitrogen dioxide levels [53, 54]. Again, this analysis did not consider the situation of social distancing and quarantine, which is important as a future analysis direction. In addition, if the vaccination rate increases and the Bangladeshi population complies with safety standards, the prediction results will be incorrect.

## 5. Conclusions

In this analysis, we used the two most popular models for forecasting epidemiology: one is mathematical approach based on SIR and the other one is a well-known machine learning algorithm, that is, the PROPHET algorithm. Again, we also applied the hierarchical *k*-means clustering approach to identify the COVID-19 hotspot for Bangladesh. In summary, we conclude that Dhaka city is the hotspot (higher risk zone) for the COVID-19 pandemic in Bangladesh based on the hierarchical *k*-means clustering approach. We conducted an assessment of the SIR model to forecast for the next 365 days based on various scenarios of COVID-19 in Bangladesh. In terms of the SIR predictive model, we revealed that confirmed and death cases were gradually decreasing in Bangladesh. Furthermore, the PROPHET algorithm predicts that all three confirmed, death, and recovered cases in Bangladesh will increase exponentially between August 2021 and August 2022. Delta variant and Omicron are still present in Bangladesh and recently vaccination rate has been lower in Bangladesh; for that reason, PROPHET algorithm is appropriate for forecasting the COVID-19 pandemic trend in Bangladesh. The government should take different actions to reduce the spread of COVID-19 by ensuring vaccination for all again by executing the law for people to eliminate all types of gathering, proper hygiene practices (mandatory wearing of masks outside home and handwashing with hand sanitizer/soap for 20 seconds), ensuring home and institutional quarantine for COVID-19 carriers, and creating a campaign for social distance by enacting rigorous lockdown procedures for this pandemic. We hope we will win this pandemic war, as our prediction suggests.
